# Endothelium in Diseased States

**DOI:** 10.1155/2014/810436

**Published:** 2014-04-30

**Authors:** Iveta Bernatova, Ramaroson Andriantsitohaina, Silvia M. Arribas, Vladimir V. Matchkov

**Affiliations:** ^1^Institute of Normal and Pathological Physiology, Slovak Academy of Sciences, Sienkiewiczova 1, 813 71 Bratislava, Slovakia; ^2^LUNAM Université, INSERM UMR U1063, Université d'Angers IBS-IRIS, rue des Capucins, 49100 Angers, France; ^3^Departamento de Fisiologia, Facultad de Medicina, Universidad Autónoma de Madrid, Arzobispo Morcillo 4, 280 29 Madrid, Spain; ^4^Department of Biomedicine, Aarhus University, Ole Worms Alle Building 4, 1163, 8000 Aarhus C, Denmark

Endothelium is the endocrine organ essential for maintenance of homeostasis in the entire body. Endothelium operates by a broad spectrum of signaling molecules controlling the contractile state of vascular smooth muscles and cardiomyocytes; long distance intercellular synchronization within the vascular wall; adhesive, coagulant, and rheological properties of blood; and permeability of the vascular wall. Dysfunctions in these signaling pathways result in loss of the important homeostatic functions as well as in engagement of the endothelium into activities leading to pathologies. This represents the two sides of the coin, where endothelium can be either a protective or “health-threatening” organ depending on the signaling involved. Endothelial dysfunction (ED) was observed in aging as well as in major lifestyle-related diseases suggesting that the endothelium can serve as a target for prevention and treatment of various diseases. Significant progress achieved in the identification of new endothelium-dependent signaling pathways and characterization of their role in various pathological states has been made since the identification of nitric oxide (NO) as an endothelium-derived relaxing factor. This special issue aims to highlight this progress and stimulate further this development. Variable functions of the endothelium are presented in six reviews, seven research articles, and two clinical studies.

The review of G. Favero et al. summarizes the endothelial function in major cardiovascular diseases and diabetes, endothelial alterations due to aging and smoking, and beneficial effects of physical activity and dietary products for endothelial function. Recent insights into the paracrine modulation of cardiomyocyte contractility by cardiac endothelial cells were reviewed by J. Noireaud and R. Andriantsitohaina, who suggested that some of the recently considered deleterious signals, like reactive oxygen species (ROS), need to be carefully reevaluated.

The review by I. Bernatova summarized a complex cross talk among the individual endothelium-derived factors and mentioned a possible classification of ED. In addition, this review analyzed the role of endothelium in the development of hypertension in various experimental models and it concluded that ED might be both a cause and the consequence of high blood pressure. Two other studies by A. Puzserova et al. and by P. Slezak et al. investigated the endothelial function in rats with genetic predispositions to hypertension, taking into account either aging in early periods of life or exposure to the chronic stress. A. Arnalich-Montiel et al. showed that short-term treatment of hypertensive rats with the *β*-blocker esmolol not only improved coronary artery remodeling but also normalized endothelium-dependent relaxation due to improved NO bioavailability and reduction of oxidative stress. S. Liskova et al. studied the influence of hypertension and aging on the norepinephrine-induced vasoconstriction mediated by Ca^2+^-dependent Cl^−^ channels.

L. Pernomian et al. demonstrated the negative effect of ROS on* Mas* receptor-mediated signaling and carotid artery flow in diabetic rats. The review by A. Magenta et al. summarized the molecular mechanisms underpinning endothelial dysfunction in diabetic patients. The authors paid special attention to an important regulator of intracellular ROS production—p66Shc protein. V. Golubinskaya et al. studied the effect of myeloperoxidase deficiency on endothelial function in mice and found that myeloperoxidase deficiency failed to potentiate endothelium-dependent relaxation.

A balance between NO and ROS in rats exposed to intermittent and continuous chronic hypoxia was studied by P. Siques et al. The authors showed that continuous hypoxia produced larger nitrosative damage compared to intermittent exposure that could be related to the larger severity of cardiovascular alterations. The role of NO in cardiovascular and hypoxia-related respiratory diseases and the involvement of asymmetric dimethylarginine and its degrading enzyme dimethylarginine dimethylaminohydrolase in development of ED were reviewed by N. Lüneburg et al. S. H. van Ierssel et al. discussed the crucial role of endothelium in the development of organ failure and secondary ischemia. Their review pointed towards possible prognostic function of endothelial progenitor cells and endothelial microparticles as biomarkers for endothelial repair and damage, respectively.

Last but not least, two clinical studies completed this special issue. Study by L. M. Cotie et al. underlined the beneficial effect of exercise and diet on endothelial function in young obese woman. Another clinical study by K. D. Currie et al. examined acute endothelial responses to exercise in patients with coronary artery disease and found its correlation with the degree of resting endothelial dysfunction.

All articles involved in this special issue had brought about new and valuable information on the role of endothelium in health and diseases.

As the guest editors of this special issue we would like to acknowledge all authors who contributed either by reviewing recent literature or by original experimental and clinical studies, making this issue valuable for diverse audience of researchers interested in the endothelium in various diseased states.

*Iveta Bernatova* *Ramaroson Andriantsitohaina* *Silvia M. Arribas* *Vladimir V. Matchkov*

